# Mumijo Traditional Medicine: Fossil Deposits from Antarctica (Chemical Composition and Beneficial Bioactivity)

**DOI:** 10.1093/ecam/nen072

**Published:** 2010-09-15

**Authors:** Anna Aiello, Ernesto Fattorusso, Marialuisa Menna, Rocco Vitalone, Heinz C. Schröder, Werner E. G. Müller

**Affiliations:** ^1^Dipartimento di Chimica delle Sostanze Naturali, Università di Napoli “Federico II”, via D. Montesano 49, 80131 Napoli, Italy; ^2^Abteilung für Angewandte Molekularbiologie, Institut für Physiologische Chemie, Johannes Gutenberg-Universität Mainz, Duesbergweg 6, 55099 Mainz, Germany

## Abstract

Mumijo is a widely used traditional medicine, especially in Russia, Altai Mountains, Mongolia, Iran Kasachstan and in Kirgistan. Mumijo preparations have been successfully used for the prevention and treatment of infectious diseases; they display immune-stimulating and antiallergic activity as well. In the present study, we investigate the chemical composition and the biomedical potential of a Mumijo(-related) product collected from the Antarctica. The yellow material originates from the snow petrels, *Pagodroma nivea*. Extensive purification and chemical analysis revealed that the fossil samples are a mixture of glycerol derivatives. *In vitro* experiments showed that the Mumijo extract caused in cortical neurons a strong neuroprotective effect against the apoptosis-inducing amyloid peptide fragment *β*-fragment 25–35 (A*β*25–35). In addition, the fraction rich in glycerol ethers/wax esters displayed a significant growth-promoting activity in permanent neuronal PC12 cells. It is concluded that this new Mumijo preparation has distinct and marked neuroprotective activity, very likely due to the content of glycerol ether derivatives.

## 1. Introduction

“Reports on the high biodiversity of marine animals date back to Aristotle (384–322 BC) [1], who gave—in his 5th book on the History of Animals—extensive descriptions on those sponge species near the island of Lesbos that have been commercially used later (reviewed in [2])”. Likewise, since Aristotle [1] the tar-like substance, of white over yellow to black color, which is used in traditional medicine, has been termed collectively Mumijo, Mumie or Mumiyo [3]. Pfolsprundt [4] also mentioned the alleviating remedy in his compendium. This traditional drug is widely distributed in Russia (termed there Mumie or Mumiyo), India (Saljit), Birma [Kao-tun (blood of the mountain)], Altai Mountains [Barachgschin (oil of the mountain)], Mongolia [Brogschaun (mountain juice)] and Iran Kasachstan, and Usbekistan as well as in Kirgistan [Arakul dshibal (mountain sweat)] [3]. The origin of the word “Mumijo” goes back to the Greek and means “saving the body”. The Asian Mumijo is found at high altitudes as deposits in walls and caves where they are embedded into rocks. These organic accumulations of unknown origin may reach weights of up to 500 kg; some are considered to be up to 3000-years old [3, 5, 6]. The chemical composition of Asian Mumijo of *∼*20% of minerals, 15% of proteins, 5% of lipids and 5% of steroids has been described in detail [3]; the rest are carbohydrates, alkaloids and amino acids. A series of medical applications has been described (reviewed in [3]), including immune-stimulating and antiallergic activity as well as an ameliorating effect against gastric and intestinal ulcers and finally healing of bone fractures. Furthermore, a protective effect against radiation and a favorable nootropic property [7] have been described.

The term Mumijo is not only restricted to the black, tar-like substance from Asia [3], but it is also used for the paleoenvironmental records—subfossil stomach oil deposits from Antarctica [8]. This material is yellow and originates from the snow petrels, *Pagodroma nivea*. The cross composition of this waxy organic material, found in petrel-breeding colonies, had been determined by Warham et al. [9]. These authors reported that the stomach oil of the Petrels consists primarily of triglycerides from which the birds obtain their energy through their intermediary metabolism. The fatty acid “oil” composition was published earlier by Lewis [10], while a more detailed analysis was given by Place et al. [11] who reported that the stomach oil of the Leach's Storm-Petrel, *Oceanodroma leucorhoa*, is composed to >90% of neutral lipids (e.g., triglycerides, wax esters and glycerol ethers). As expected, the composition of these organic ingredients is dynamic. The amount of deposition of the oil is depends on the environmental living conditions of the birds; Warham [12] underscored also the ecological importance of the stomach oil for the seasonal requirements of the animals. However, a state-of-the-art analysis especially of the fossil deposits is missing. The material, investigated in the present contribution was collected during the “GeoMaud”—Geoscientific Expedition to Dronning Maud Land (Antarctica) (http://www.bgr.bund.de/cln_011/nn_322990/DE/Themen/MeerPolar/Polarforsch-ung/Projekte/Antarktis_Projekte/GEOMAUD.html) during expeditions between November 1, 1995 and August 25, 2005. The yellow stony Mumijo material was collected from the Schirmacher Oasis (11°35′E, 70°45′S) as described [13, 14] and determined to be *∼*3000-years old. One reason for the intense study also of the antarctic Mumijo is its value as palaeoclimate biomarker [8]. Especially for the Late Quaternary paleoenvironmental history, this material is suitable to obtain further information about the climate changes and the local ice retreats, moraines and Petrel occupation history. The layers of fossil stomach oil can become 50 cm thick and are deposited only on ice- and snow-free locations. The deposits are indicative for the breeding places of the Petrels and can hence give answers to paleoclimate-related questions, for example, the retreat of glaciers.

In the present study, we report about the chemical composition of the fossil sample of Mumijo as well as about its neuroprotective and cell growth stimulatory effects. Our results correlate this latter activity particularly to the presence of *α*-glyceryl ethers in this material. However, it cannot be ruled out that Mumijo causes, as a complex formulation, in addition also an amelioration of a series of afflictions and may act also as an antimicrobial, antiviral, antitumor, antiallergic, immunomodulating or anti-inflammatory medicine, similar to the active compounds from mushrooms [15], or of Propolis [16], or “Kampo” compounds [17] as well as of Arabic medical herbs [18].

## 2. Materials and Methods

### 2.1. Materials

Alzheimer-*β* fragment [A*β*25–35] (A 4559), 3-(4,5-dimethylthiazol-2-yl)-2,5-diphenyltetrazolium bromide (thiazolyl blue; MTT; M 2128), as well as additional chemical substances were obtained from Sigma (St Louis, MO, USA).

### 2.2. Instruments

Electrospray ionization (ESI) mass spectra were obtained on an API 2000 mass spectrometer. Nuclear magnetic resonance (NMR) experiments were performed on a Varian Unity INOVA 500 spectrometer; chemical shifts refer to the residual solvent signal (CD_3_OD: **δ**_H_ = 3.31, **δ**_C_ = 49.0; CDCl_3_: **δ**_H_ = 7.26; **δ**_C_ = 77.0). Medium-pressure liquid chromatographic (MPLC) analyses were carried out on a Büchi 861 apparatus with SiO_2_ (230–400 mesh) packed columns. High-performance liquid chromatography (HPLC) separations were achieved on a Knauer 501 apparatus equipped with an RI detector. GC-MS spectra were performed with a Hewlett- Packard 5890 gas chromatograph equipped with a split/splitness injector and connected to a Mass Selective Detector (MSD) HP 5970 MS using electron impact ionization (EI) at a ionization energy of 70 eV. HPLC was achieved with a Varian Prostar 210 apparatus equipped with a Varian 350 refractive index detector or a Varian 325 UV detector.

### 2.3. Mumijo

**Figure 1:**
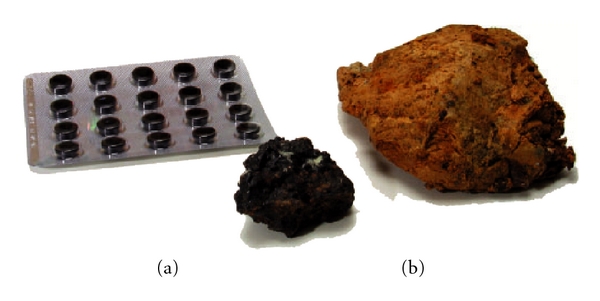
Mumijo samples. (a) Mumijo from Samarkand (Turkestan) (black). (b) Mumijo from Antarctica (yellow). In addition, the extract used in traditional formulation as medicine in Russia (in the background). (a) Mumijo Altai; (b) Mumijo Panacea.

The material was obtained from Dr Ulrich Wand (Alfred-Wegener-Institut Bremerhaven) and collected during the “GeoMaud”—Geoscientific Expedition to Dronning Maud Land (Antarctica) (http://www.bgr.bund.de/cln_011/nn_322990/DE/Themen/MeerPolar/Polarforsch-ung/Projekte/Antarktis__Projekte/GEOMAUD.html) November 1, 1995 to August 25, 2005. The location had been the Schirmacher Oasis (11°35′E; 70°45′S). The yellow stony material from Antarctica (Figure 1(b)) is compared with the brownish tar-like deposits, which had been obtained from Samarkand (Turkestan) (Figure 1(a)).

### 2.4. Extraction and Isolation

A first Mumijo extract was obtained by grinding the Antarctic material in a mortar and suspending it in dimethyl sulfoxide. After shaking for 24 h at 4°C the clear extract was obtained by centrifugation (5000 g; 10 min; 4°C). The concentration cited under Results section refers to the amount of solid Mumijo used for extraction.

For the chemical analysis, the fossil material (8.5 g dry weight after extraction) was homogenized and extracted first with methanol (3 × 300 ml) and then with chloroform (3 × 200 ml). Combined extracts were concentrated *in vacuo* and a crude extract (5.8 g) was obtained. This was subjected to fractionation by silica gel MPLC to give six fractions, A to F, using hexane, EtOAc and MeOH as a progressively polar solvent system series. All fractions were subjected to a preliminary spectroscopic inspection (^1^H NMR, ESIMS). Fractions B to D were showed to be neutral lipid mixtures and were subsequently separated and/or analyzed, as indicated.

Fraction B (4 g) contained wax esters, whose fatty acid and alcohol compositions were determined by GC-MS in their natural state. GC-MS analysis was performed on a fused silica column (25 m × 0.20 mm HP-5; cross-linked 25% Ph Me silicone; 0.33-mm film thickness). The oven temperature was programmed from 150°C to 350°C at a rate of 10°C/min and held at the final temperature for 10 min. Helium was used as carrier gas at a constant flow rate of 1.0 ml/min and a gas inlet pressure of 13.3 psi. Quantitative determination was based on the area of GLC peaks (Table 1). *Wax esters* (*fraction B*): ^1^H NMR (CDCl_3_): **δ** 0.86 (t, *J* = 6.6 Hz, 6 H), 1.23 (broad signal, alkyl chain protons), 1.52–1.58 (br., 4 H), 2.26 (t, *J* = 7.5 Hz, 2 H), 4.03 (t, *J* = 6.7 Hz, 2 H) p.p.m.

Fraction C (1.1 g) was shown to be a mixture of fatty acids. These had been methylated with diazomethane and the resulting esters were analyzed by GC-MS analyses on a fused silica column as above. The temperature of the column was changed 5 min after injection from 150°C to 300°C with a slope of 5°C/min. The quantitative determination was based on the area of GLC peaks and the results of the analysis are summarized in Table 2. *Fatty acid methyl esters* (*fraction C*):  ^1^H NMR (CDCl_3_): **δ** 0.85 (t, *J* = 6.6 Hz, 3 H), 1.23 (broad signal, alkyl chain protons), 1.58 (m, 2 H), 2.30 (t, *J* = 7.5 Hz, 2 H), 3.65 (s, 3 H) p.p.m.

Fraction D (0.4 g) was re-chromatographed on an RP-18 column by MPLC (H_2_O → MeOH → CHCl_3_), thus giving a monoglycerides fraction (160 mg, Fraction D/2) eluted with H_2_O/MeOH 2:8, and a monoalkyl glycerol ethers fraction (90 mg, Fraction D/3) eluted with 100% MeOH. An aliquot each of fractions D/2 and D/3 (20 mg each) was dissolved in pyridine (500 *μ*l) and allowed to react with Ac_2_O (200 *μ*l) for 12 h. The reaction mixtures were concentrated and the residues were purified by normal phase HPLC (Luna Silica 5 *μ*m, 250 × 4.60 mm, hexane/AcOEt 9:1 as the eluent). *Monoglyceride diacetates from fraction D/2:* ^1^H NMR (CDCl_3_): **δ** 0.86 (t, *J* = 6.7 Hz, 3 H), 1.23 (broad signal, alkyl chain protons), 2.04 (m, 4 H), 2.06 (s, 3 H), 2.07 (s, 3 H), 2.30 (t, *J* = 7.5 2 H), 4.11–4.16 (overlapped signals, 2 H), 4.25–4.31 (overlapped signals, 2 H) 5.23 (m, 1 H), 5.33 (m, 2 H) p.p.m. ESI (positive ion mode): *m/z*: 407, 409, 435, 437, 463, 465, 491, 493, 521, 549 [M + Na]^+^ series. The quantitative estimation, reported in Table 2, is based on the relative intensity of the peaks. *Glyceryl ethers diacetates from fraction D/*3: ^1^H NMR (CDCl_3_): **δ** = 0.86 (t, *J* = 6.6 Hz, 3 H), 1.23 (broad signal, alkyl chain protons), 1.54 (m, 2 H), 2.04 (m, 4 H), 2.05 (s, 3 H), 2.07 (s, 3 H), 3.41 (m, 2 H), 3.51 (d, *J* = 5.2, 2 H), 4.14 (dd, *J* = 12.0, 6.4 Hz, 1 H) 4.31 (dd, *J* = 12.0, 3.6 Hz, 1 H), 5.16 (m, 1 H), 5.33 (m, 2 H) p.p.m. ESI (positive ion mode): *m/z*: 393, 395, 421, 423, 449, 451, 477, 479, 507, 535 [M + Na]^+^ series.

### 2.5. Cell Culture: Cortical Cells

Primary cortical cells were prepared according to a modified procedure [19, 20] from the brains of 19-day-old Wistar rat embryos by dissociation (0.025% trypsin in Hanks' balanced salt solution without Ca^2+^ and Mg^2+^). The cell suspension was centrifuged and the pellet was resuspended in Dulbecco's modified Eagle's medium (4500 mg of glucose/l), supplemented with 100 mU/l insulin, 2 mM glutamate and 10% fetal calf serum. After incubation for 48 h in poly-l-lysine-coated plastic 96-well plates, the medium was supplemented with 10 *μ*M uridine, 10 *μ*M fluorodeoxyuridine and 1 *μ*M cytosine arabinofuranoside (to eliminate proliferating non-neuronal cells) for 3 days. The cultures contained >85% neurons; the other cells were glial fibrillary acidic protein-positive [20, 21]. Cells were routinely exposed to the A*β* fragment A*β*25–35 at a concentration of 1 *μ*M for 5 days. The A*β*25–35 was prepared in a stock solution of 900 *μ*M in distilled water and stored for 5 days at 4°C before use. Mumijo extract was added at the indicated concentrations 2 h before incubation of the cells with A*β*25–35.

### 2.6. Cell Culture: PC12

In parallel, the extracts were tested in the permanent PC12 cell line that is derived from pheochromocytoma of the rat adrenal medulla. The tumor cells were grown as described earlier [22], but with the modification that RPMI-1640 medium, enriched with 10% fetal calf serum, was used. All cells were kept in a humidified atmosphere of 5% CO_2_ and 95% air. Cells were seeded in 96-well plates at a density of 5 × 10^3^ cells per well with or without the extracts. After 72 h, plates were analyzed on a microplate reader and the ED_50_ concentrations were determined [23].

### 2.7. Evaluation of Viable Cells

The viability of total cells was determined with the MTT colorimetric assay system [24], followed by evaluation with an ELISA plate reader (Bio-Rad model 3550, equipped with the program NCIMR IIIB). Ten parallel assays were performed for each concentration of the respective extracts. The results were analyzed by paired Student's *t*-test [23].

## 3. Results

### 3.1. Chemical Analysis of Mumijo Extract

The wax esters fraction was analyzed by GC-MS using EI, according to the method proposed by Reiter et al. [25]. This rapid method allows to analyze the composition of a wax in its natural state and to obtain a reliable and complete profile of wax esters. EI spectra of wax esters [R^1^COOR^2^] contain a single molecular ion [M]^+^ alongside a set of dominant ions [R^1^CO_2_H_2_]^+^ deriving from a double hydrogen rearrangement fragmentation at the ester group. These ions show a difference of 28 amu and lead to the conclusion that the individual GC peaks contain wax ester isomers with the same carbon number and same degree of unsaturation, but different position of the esters moiety within the wax ester due to different chain lengths of carbonyl- and ester components. In our case, due to the relatively high abundance of [R^1^CO_2_H_2_]^+^ ions, the assignment of the individual was esters isomers was possible, as shown in Table 1; further evidence for our results was provided by the presence of [R^2^-H]^+^ ions and [R^1^CO]^+^ acylium ions.

Fractions D/2 and D/3 were shown (by NMR) to be a mixture of glycerol derivatives; the identification of their components was carried out by NMR and ESIMS. An aliquot each of fractions D/2 and D/3 was acetylated by treatment with acetic anhydride and pyridine and then purified by normal phase HPLC. The fractions turned out to be mixtures of monoglyceride diacetates (fraction D/2) and glyceryl ethers diacetates (fraction D/3) by NMR analysis (see Section 2). A confirmation was also achieved by comparing their spectroscopic properties with those reported in literature [26, 27]. The ESI mass spectra (positive ion mode) of fractions D/2 and D/3 showed a [M + Na]^+^ peak series, indicating their composition of homologs; the high field region of the ^1^H-NMR spectra of both fractions revealed that all the homologs were unbranched. The assessment of fatty acid and alcohol composition of monoglycerides and monoalkyl glycerol ethers diacetates, reported in Table 2, was based on the estimation of the relative intensity of the peaks in their ESI spectra. Table 2 shows also the free fatty acid composition determined by GC-MS analysis performed on their methyl esters.

### 3.2. Mumijo Extract Protects Cortical Neurons against A*β*25–35-Caused Reduction of Cell Viability

**Figure 2:**
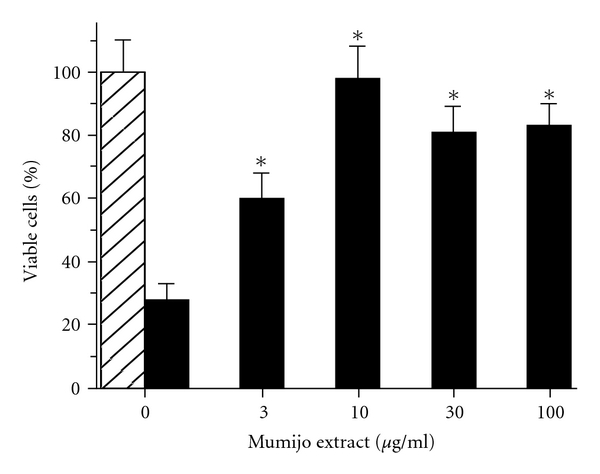
Effect of Mumijo extract on A*β*25–35-induced cell toxicity. Neurons have been treated with 1 *μ*M of A*β*25–35 for 5 days. During this period the viability of the cells dropped from 100% (hatched bar) to 28% (solid black bar) if no Mumijo extract had been added. However, if the cultures had been pre-incubated with increasing concentrations of Mumijo extract (3–100 *μ*g/ml) the *β*25–35-induced cell toxicity is reduced. Control values are set to 100% (hatched bar); *n* = 10. The means ± SEM are given. *P < .001 [versus controls (plus A*β* 25–35)]. Cell viability was determined applying the MTT assay procedure.

The toxic effect of the A*β* fragment, A*β*25–35, was assessed in primary rat cortical neurons. Application of the fragment at a concentration of 1 *μ*M caused within the 5-day incubation period a significant reduction of viable cells to 27.8 ± 6.1% *(P <* .001). Mumijo extract alone was found to have no effect on the viability of the neurons. However, if the neurons were pre-incubated with Mumijo extract prior to addition of the A*β*25–35, a significant higher cell viability was determined (Figure 2). At concentrations of 3 *μ*g/ml or higher of Mumijo extract, the percentage of viable cells increased from 27.8 ± 6.1% (in the absence of extract) to 98.6 ± 9.3% (10 *μ*g/ml) and 82.4 ± 8.9% (30 *μ*g/ml) (*P* < .001), respectively. The neuroprotective effect displayed by the Mumijo extract was still significant at 1 *μ*g/ml (not shown).

### 3.3. Mumijo Extract Promotes PC12 Cell Growth

**Figure 3:**
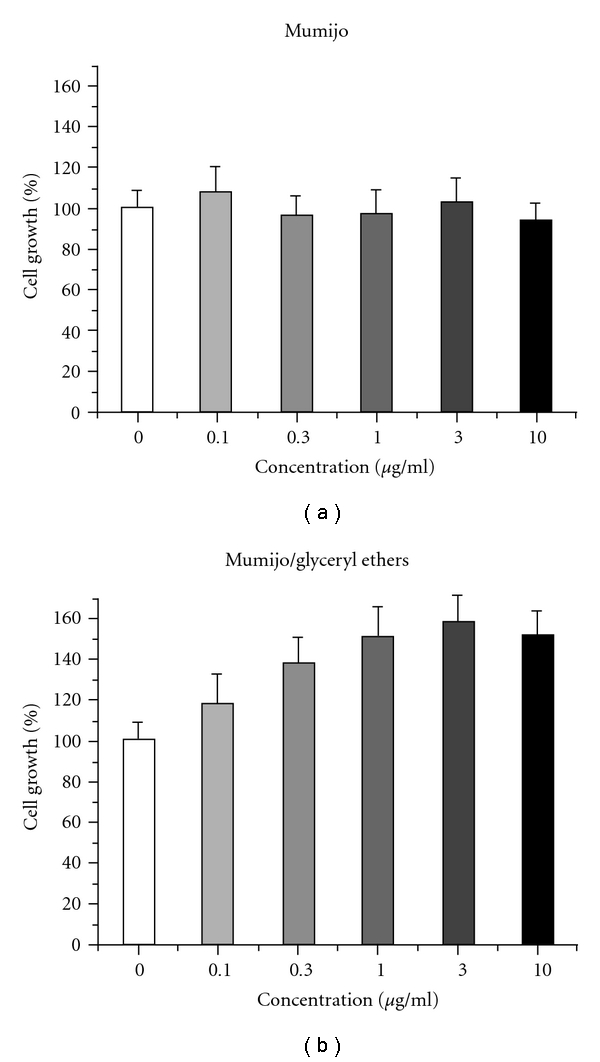
Effect of Mumijo on the cell growth of neuronal PC12 cells. (a) Effect of non-purified Mumijo extract on growth of permanent PC12 cells. (b) Fraction D/3, containing glyceryl ether diacetates caused a dose-dependent stimulation of proliferation. Incubation conditions are given under Section 2.

The permanent PC12 cell line, a model system for neuronal differentiation [28], was used as a second cell system to assess the biological activity of Mumijo (Figure 3). The non-purified extract displaced no significant growth stimulatory effect between 0.1 and 10 *μ*g/ml. However, after purification, Mumijo fraction D/3 was effective and resulted in a significant stimulation of cell growth. Already at a concentration of 0.3 *μ*g/ml, a significant increase in the growth stimulatory activity could be measured (114.0 ± 6.8%; *P* < .001), while the maximal growth promoting function was determined between 3.0 and 10.0 *μ*g/ml (139.2 ± 12.3% or 129.2 ± 10.3%, resp.).

## 4. Discussion

A detailed description of the components in Mumijo from Central Asia revealed [3] primarily inorganic components, for example, minerals (18–20%), considerable amounts of organic components, primarily of proteins (13–17%), steroids (3.3–6.5%), carbohydrates (1.5–2%) and nitrogen-containing compounds (0.05–0.08%), in addition to lipids (4–4.5%). By our activity-guided isolation procedure, using neuronal cells, we identified that the major organic, bioactive components are wax esters. The mineral content of the Antartican Mumijo has not yet been determined, leaving room also for a potential application in the treatment of bone diseases [29]. Likewise the potential biomedical activity of the monoglycerides, known to possess potent antimicrobial/microbicidal activity [30], and of the neutral glyceroglucolipids [31], comprising anti-stomach ache effectiveness, are not addressed here.

The chemical analysis of the fossil sample of Mumijo actually revealed that its composition parallels those previously reported for other samples of non-fossil material, with some substantial differences. The organic extract of Antarctic Mumijo contained mainly wax esters (70% wt), with considerable amounts of free fatty acids (20% wt). Monoglycerides and free monoalkyl glycerol ethers were also detected in significant amounts (3% wt and 1.6% wt, resp.). Monoalkyl glycerol ethers are most frequently found as alkyldiacylglycerols (similar to triacylglycerols). However, these compounds, whose occurrence in petrel stomach oils has been reported [9, 10, 31, 32], as well as triglycerides and/or diglycerides, present in large amounts in other previously examined oil samples, were not detected at all in the Mumijo sample investigated. This difference might be ascribed to the age of the sample and, as a consequence, to the effect of a slow lipolysis. Our sample also lacked cholesterol esters, found in some other Mumijo oils.

Glyceryl ethers were identified by Tsujimoto and Toyama [33] in the fraction of some fish liver oils and, subsequently, they have been found in diverse sources, including most of the petrel stomach oils investigated [10, 12]. The major monoalkyl ethers are: 1-*O*-hexadecylglycerol (16:0 alkyl or chimyl alcohol), 1-*O*-octadecylglycerol (18:0 alkyl or batyl alcohol) and 1-*O*-octadec-9-enyl glycerol (18:1 alk-9-enyl or selachyl alcohol). The trivial names go back to the fish species from which they have originally been isolated. In 1948, Berger [34] reported the central depressant action of *α*-substituted glycerol ethers, and of chimyl and batyl alcohols. Since then, reports have appeared claiming a number of further pharmacological activities, such as antimicrobial [35], tubercolostatic [36] and Lactobacillus growth-promoting activities [37], as well as a protective effect against radiation sickness [38] and radiation-induced leucopenia [37]. Bodman and Maisin [39] reported that topical application of *α*-glyceryl ethers as well as of batyl and selachyl alcohols significantly accelerated the rate of wound healing in man when the healing process had been pathologically inhibited. Burford and Gowdey [40] claimed that batyl and selachyl alcohols showed anti-inflammatory effects comparable to hydrocortisone in rats when administered p.o. In 1972, Ando et al. [41] reported the antitumor activity of fatty alcohols and *α*-glyceryl ethers of fatty alcohols. These properties could explain some medical applications of Mumijo in oriental medicine, such as its use for gastric and intestinal ulcers, healing of fractures, burns and skin diseases, tuberculosis, respiratory disease and inflammations [3].

The present finding that Mumijo is rich in (*α*-glyceryl ethers of) fatty alcohols is in accordance with recent data, which demonstrate that distinct fatty alcohols present strong potential for the treatment of neurological diseases, and are able to modulate neuroinflammation via induction of differentiation of neural stem cells into mature neurons. Based on these data, it had been proposed that those compounds might represent an approach for the treatment (or cure) of neuropathies [42]. Very well established is also the neuroprotective action of polyunsaturated fatty acids in Parkinson's as well as in Alzheimer's disease [43]. In addition, since >50 years shark liver oil has been used as a therapeutic and preventive agent. In this preparation, the most active ingredients are the ether-linked glycerols, which have been suspected to act via activation of protein kinase C resulting in a immunostimulating action on the macrophage [44].

**Figure 4:**
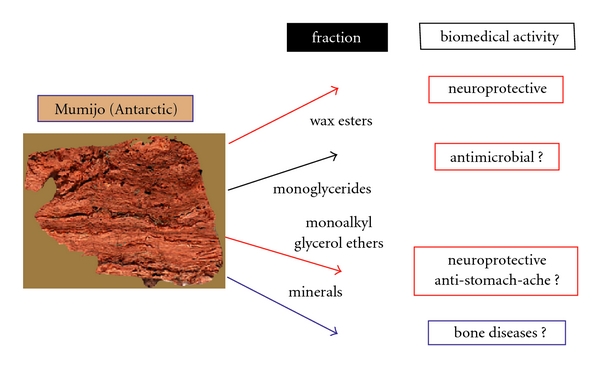
Main biomedical activity (established as well as expected from literature data) of the different organic fractions, which have been separated from Antarctic Mumijo. A further potential can be supposed from the inorganic component(s), the minerals, with respect to their ameliorating function in bone diseases. The scheme shows also a cross section through a Mumijo sample from Antarctica (size: 2.5 cm). The layered deposition of the waxy organic material is self-evident.

Taken together, our data presented here show that the Antarctic Mumijo is rich in glyceryl ether derivatives which—according to the data given—display distinct and marked neuroprotective activity. Schematically, the biomedical potential of Antarctic Mumijo is summarized in Figure 4. The main organic components, the wax esters and the glycerol ethers, are known to display neuroprotective potential. Future studies will prove if the monoalkyl ethers display also anti-stomach ache capacity. Finally, the triglycerides have to be studied for their putative antimicrobial activity. The inorganic component(s), the minerals existing in Mumijo, may have their ameliorating function in bone diseases. An outline of the exploitation strategies for traditional and modern drugs applicable in the biomedicine has been given for the Mediterranean region [45].

## Figures and Tables

**Table 1:** Wax esters in fossil stomach oil—isomer composition, content and fragments.

Total carbon atoms of wax esters chains [R^1^COOR^2^]	[M]^+^	[R^1^CO_2_H_2_]^+^	Intensity [R^1^CO_2_H_2_]^+^ (%)	[R^1^CO]^+^	[R^2^−1]^a^	Acid moiety	Alcohol moiety
28	424	229	92.3	211	196	C14	C14
	424	285	7.7	267	140	C18	C10
30	452	229	83.6	211	224	C14	C16
	452	257	15.5	239	196	C16	C14
	452	285	0.90	267	252	C18	C12
32	480	257	91.3	239	210	C16	C16
	480	229	5.9	211	238	C14	C18
	480	285	2.8	267	(182)	C18	C14
34	508	285	55.8	267	224	C18	C16
	508	257	44.2	239	(252)	C16	C18

^a^Fragments in brackets are not visible in the spectrum.

**Table 2:** Fatty acid composition of monoglycerides and free fatty acids fraction, and fatty alcohol composition of monoalkyl glyceryl ethers.

	Monoglycerides^a^	Glyceryl ethers^a^	Free fatty acids^b^
14 : 0	18.7	17.0	21.3
14 : 1	0.6	0.4	0.4
16 : 0	44.3	48.7	36.0
16 : 1	2.3	0.7	7.8
18 : 0	5.7	6.6	6.4
18 : 1	12.1	2.2	13.6
20 : 0	2.6	3.7	5.3
20 : 1	2.3	2.3	3.3
22 : 0	6.2	10.4	3.0
22 : 1	3.0	1.1	1.8
24 : 0	1.1	0.5	0.9
24 : 1	0.7	6.4	0.2

^a^Quantitative estimation (%) was based on the relative intensity of the peaks in the ESI mass spectrum.

^b^Quantitative determination (%) was based on the area of GLC peaks.
